# Extracellular nicotinamide phosphoribosyltransferase: role in disease pathophysiology and as a biomarker

**DOI:** 10.3389/fimmu.2023.1268756

**Published:** 2023-10-17

**Authors:** Elise Semerena, Alessio Nencioni, Krzysztof Masternak

**Affiliations:** ^1^ Light Chain Bioscience - Novimmune SA, Plan-les-Ouates, Switzerland; ^2^ Department of Internal Medicine and Medical Specialties, University of Genoa, Genoa, Italy; ^3^ Ospedale Policlinico San Martino IRCCS, Genoa, Italy

**Keywords:** eNAMPT, adipocytokine, inflammation, metabolic diseases, cancer

## Abstract

Nicotinamide phosphoribosyltransferase (NAMPT) plays a central role in mammalian cell metabolism by contributing to nicotinamide adenine dinucleotide biosynthesis. However, NAMPT activity is not limited to the intracellular compartment, as once secreted, the protein accomplishes diverse functions in the extracellular space. Extracellular NAMPT (eNAMPT, also called visfatin or pre-B-cell colony enhancing factor) has been shown to possess adipocytokine, pro-inflammatory, and pro-angiogenic activities. Numerous studies have reported the association between elevated levels of circulating eNAMPT and various inflammatory and metabolic disorders such as obesity, diabetes, atherosclerosis, arthritis, inflammatory bowel disease, lung injury and cancer. In this review, we summarize the current state of knowledge on eNAMPT biology, proposed roles in disease pathogenesis, and its potential as a disease biomarker. We also briefly discuss the emerging therapeutic approaches for eNAMPT inhibition.

## 1 Introduction

Nicotinamide phosphoribosyltransferase (NAMPT) is a homodimeric class II phosphoribosyltransferase (EC 2.4.2.12) ubiquitously expressed in mammalian tissues and playing a crucial role in nicotinamide adenine dinucleotide (NAD) metabolism (1, 2). NAMPT is the rate-limiting enzyme of the NAD salvage pathway, which catalyzes the production of nicotinamide mononucleotide (NMN) from nicotinamide (NAM) and 5-phosphoribosyl-1-pyrophosphate (PRPP) (3). NMN is converted to oxidized NAD (4), which then fuels cellular redox reactions and the activity of NAD-degrading enzymes, such as the cluster of differentiation (CD) 38 (CD38), poly (ADP-ribose) polymerases (PARPs), and sirtuins (SIRTs 1–7) (1, 5). These NAD-dependent enzymes mediate fundamental intracellular processes, including cell signaling, DNA repair, apoptosis and adaptive responses to cell stressors (1, 3, 5). Thus, by modulating NAD levels, NAMPT plays a pivotal role in cell metabolism and survival (3). The *NAMPT* gene is highly conserved (6–8), and orthologs of the mammalian *NAMPT* are found not only in other vertebrates [e.g., birds (8) and fish (9)], but even in sponges (9) and bacteria (10). The NAMPT enzyme is found both in the nucleus and in the cytosol, at varying levels, depending on the cell cycle phase (11, 12). Some studies have suggested that NAMPT may also be localized in mitochondria (13–15). In addition, Yoshida et al. demonstrated that NAMPT is transported through the systemic circulation via extracellular vesicles (EVs), before being reinternalized to participate in NAD production in recipient cells (16).

As mentioned above, NAMPT is not only found inside cells and EVs, but can also be directly secreted into the extracellular space. Historically, diverse names were attributed to extracellular NAMPT (eNAMPT), as its diverse functions were being characterized. eNAMPT was first identified as a cytokine secreted by pre-B cells and named “pre-B-cell colony enhancing factor” (PBEF) due to its ability to synergize with interleukin (IL)-7 and stem cell factor (SCF) in promoting pre-B-cell colony formation (17). eNAMPT was also baptized “visfatin”, reflecting its adipokine functions and its secretion from visceral adipose tissue (7, 18). Eventually, NAMPT was found to be released by all cellular types (2), and upregulated in various types of metabolic and inflammatory disorders (19–21). The present publication reviews relevant papers focusing on the biology of eNAMPT, its role as a biomarker and its contribution to disease pathogenesis. The emerging eNAMPT-targeting therapies are also examined. The references gathered in this review were found in PubMed/MEDLINE using “eNAMPT”, “visfatin” or “PBEF” as keywords. We included original and review articles published before July 2023.

## 2 NAMPT is released into extracellular space

As mentioned above, NAMPT is released by a wide range of cell types, including adipocytes, β-cells, immune cells, neurons, endothelial cells, cardiomyocytes and cancer cells (reviewed in Grolla et al. (2)). NAMPT secretion is an active phenomenon and is not a consequence of cell lysis or cell death (18, 22, 23). In healthy individuals, NAMPT secretion follows a diurnal rhythm, peaking in the afternoon (24). eNAMPT was found in serum (3), but also in synovial fluid (25), follicular fluid (26), bronchoalveolar lavage (BAL) fluid (27), saliva (28), cerebrospinal fluid (29) and stools (30).

As reviewed in Carbone et al. (3), the different stimuli promoting NAMPT secretion can be grouped into three main categories: 1) cellular stress [e.g., ischemia, oxygen-glucose deprivation, oxidative and endoplasmic reticulum [ER] stress, hypoxia (31)]; 2) nutritional cues (e.g., glucose or insulin); and 3) inflammatory signals [e.g., lipopolysaccharide [LPS], tumor necrosis factor-α [TNF-α], IL-1β, interferon-γ [IFN-γ] (32)]. However, the exact mechanism of NAMPT release into the extracellular space remains unclear, as eNAMPT lacks the peptide signal for secretion through the classical ER-Golgi secretory pathway (17). Furthermore, no canonical caspase-1 cleavage site is present in the *NAMPT* gene sequence (9), which suggests that NAMPT is not released via the conventional secretory pathway. In general, NAMPT secretion was found not to be affected by inhibitors of the ER-Golgi pathway such as brefeldin A or monensin (18, 32–35). Further studies are needed to clarify the mechanisms of NAMPT release into the extracellular space.

Under non-stimulating conditions, eNAMPT only accounts for 1% of total NAMPT protein (31). As compared to intracellular NAMPT, eNAMPT bears some distinctive post-translational modifications, such as different acetylation levels. Namely, SIRT-1-mediated deacetylation of NAMPT on lysine 53 was shown to facilitate its release (36). On the contrary, deacetylation of NAMPT by SIRT-6 reduced NAMPT secretion (37).

## 3 eNAMPT, a factor with multiple functions

**Figure 1:**
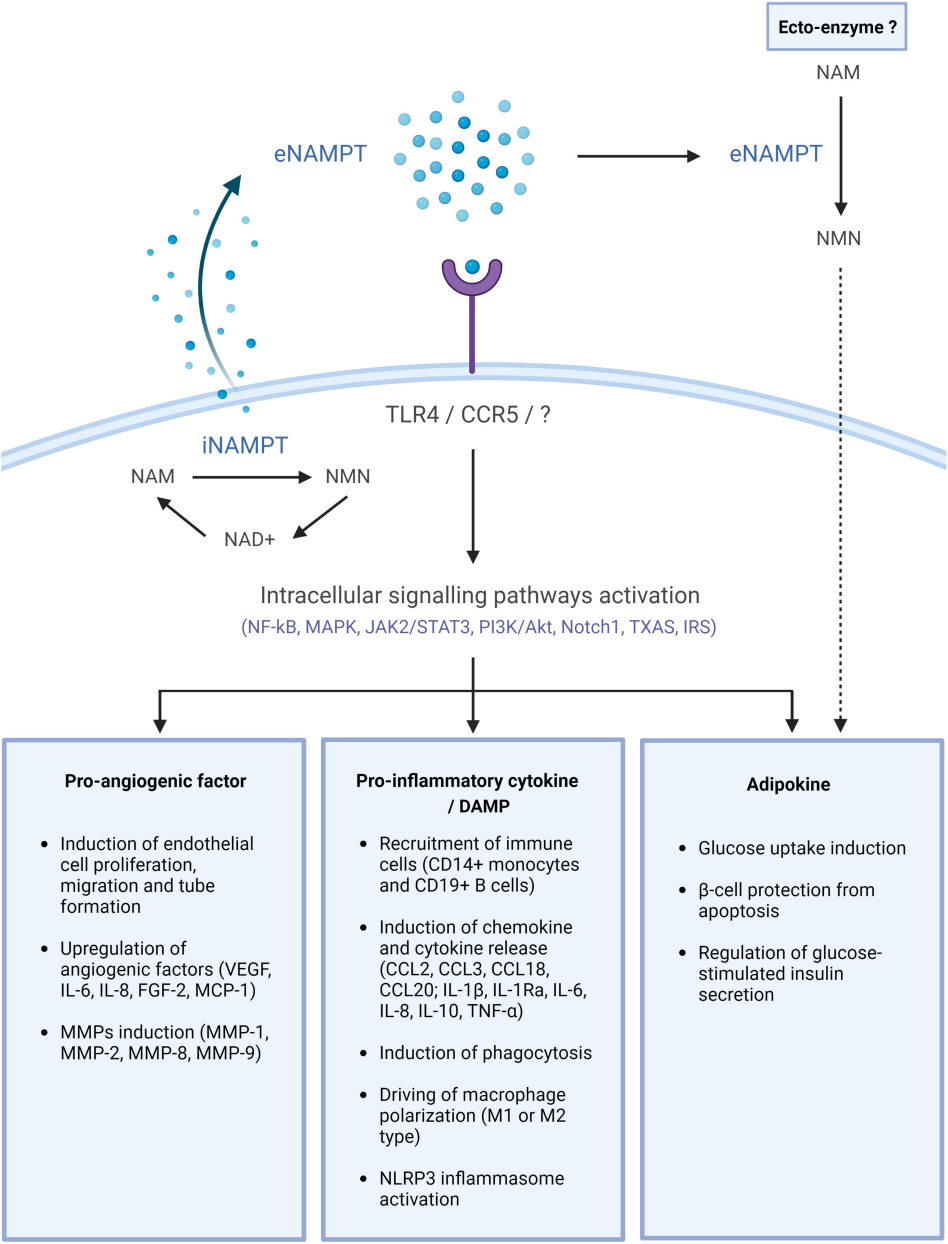
eNAMPT, a DAMP/adipocytokine with multiple functions. NAMPT is released into the extracellular space through an unknown mechanism. There, it may bind cell surface receptors, including TLR4 or CCR5, and activate intracellular signaling pathways, eventually leading to the initiation of diverse physiological and/or pathological processes. eNAMPT may possibly also act as an ectoenzyme in the extracellular space, generating NMN, which can subsequently be internalized and participate in intracellular NAD biosynthesis. This image was created with BioRender.com. Akt, protein kinase B; CD, cluster of differentiation; CCL, chemokine (C-C motif) ligand; eNAMPT, extracellular nicotinamide phosphoribosyltransferase; FGF, fibroblast growth factor; IL, interleukin; IR, insulin receptor; IRS, insulin receptor substrate; JAK, Janus kinase; MAPK, mitogen-activated protein kinases; MCP-1, monocyte chemoattractant protein-1; MMP, matrix metalloproteinase; NAD, nicotinamide adenine dinucleotide; NAM, nicotinamide; NF-κB, nuclear factor kappa-light-chain-enhancer of activated B cells; NMN, nicotinamide mononucleotide; NLRP3, NLR family pyrin domain containing 3; Nocht1, neurogenic locus notch homolog protein 1; PI3K, phosphatidylinositol-3-kinase; STAT3, signal transducer and activator of transcription 3; TXAS, thromboxane synthase; TNF, tumor necrosis factor; VEGF, vascular endothelial growth factor.

Numerous and diverse functions have been reported for eNAMPT and they were associated with its enzymatic, pro-inflammatory, pro-angiogenic and adipocytokine activities. These are described in the paragraphs below and summarized in **Figure 1**.

### 3.1 eNAMPT as ectoenzyme

It is not clear yet whether eNAMPT shows biologically relevant phosphoribosyltransferase activity in the extracellular milieu. NAMPT dimerization is essential for enzymatic activity, as shown by the reduced activity of non-dimerizing S199D and S200D *NAMPT* mutants (18, 38). Various studies have reported that eNAMPT, like its intracellular form, is also a homodimer (18, 34, 39, 40), suggesting that it may be enzymatically active in the extracellular space. In support of this, Revollo et al. demonstrated that adipocyte-derived eNAMPT exerts a strong NAD biosynthetic activity, which is even higher than that of intracellular NAMPT; the authors also found high levels of NMN in mouse plasma (18). eNAMPT has also been shown to increase extracellular NMN and NAD levels in vascular smooth muscle cell cultures and MCF7 cell cultured medium (41, 42). Furthermore, studies show that the extracellular NMN may enter cells and contribute to intracellular NAD biosynthesis (42–44).

On the other hand, the NAMPT substrates, NAM and PRPP, are present at low concentrations or are virtually absent from the extracellular milieu under physiological conditions (45). The same for ATP, which is required for the next enzymatic step, the conversion of NMN to NAD (1, 45). This implies that eNAMPT probably lacks sufficient substrates required for a sustained and functionally relevant enzymatic activity outside the cells. Nonetheless, certain pathological conditions could present a more favorable milieu for NAMPT enzymatic activity. For instance, the tumor microenvironment is characterized by hypoxic areas with elevated rates of necrosis and an acidic pH, and could thus bear increased levels of eNAMPT substrates (41).

### 3.2 eNAMPT as adipocytokine

Adipocytokines are molecules released from adipose tissues, which are involved in the regulation of glucose homeostasis, body weight, inflammation, blood pressure and tumorigenesis (46). The first description of eNAMPT as an adipocytokine was made in 2005 by Fukuhara et al. (47), demonstrating that eNAMPT is secreted by mouse adipocytes *in vitro*. This was subsequently confirmed in rats, where eNAMPT production was reported in perivascular adipose tissues (42). In addition, eNAMPT was shown to be expressed in human visceral, subcutaneous, and epicardial fat tissues (47–49). Fukuhara et al. (47) also showed that, similarly to insulin, eNAMPT lowers glucose levels in cultured adipocyte cells and *in vivo* by directly activating the insulin receptor (IR), but the publication was eventually retracted due to a lack of reproducibility of eNAMPT to IR binding experiments (50). Some subsequent studies have confirmed the insulin-mimetic effects of eNAMPT in human osteoblasts (51) and glomerular mesangial cells (52). Consistent with this, eNAMPT-mediated phosphorylation of IR (51, 53) and of the insulin receptor substrates (IRS)-1 and IRS-2 (51) was demonstrated in human osteoblasts and in mouse pancreatic β-cells. Yet another study reported that, to the contrary, eNAMPT-mediated increase in glucose transport did not involve IRS-1 phosphorylation in skeletal muscle (54). Thus, the interaction of eNAMPT with the IR signaling pathway remains unclear and highly controversial.

eNAMPT may also play a vital role in maintaining the viability and the function of pancreatic β-cells, since it was shown to prevent apoptosis and free-fatty-acid-induced metabolic dysfunction in the MIN6 pancreatic cell line (55). In line with that, Revollo et al. demonstrated that eNAMPT mediated the regulation of glucose-induced insulin release by β-cells (18). Of note, they showed that the enzymatic activity, rather than insulin-mimetic activity of eNAMPT is key for this process, supporting the notion that eNAMPT biosynthetic activity and its product, NMN, directly or indirectly maintain β-cell function (56, 57). Another study revealed that exogenous administration of NMN to mice on a high-fat diet (HFD) improved their impaired glucose tolerance and lipid profile by restoring normal NAD levels in white adipose tissue (WAT) and the liver (58).

### 3.3 eNAMPT as a mediator of inflammation

The pro-inflammatory activity of eNAMPT is well documented. Numerous studies have demonstrated that eNAMPT activates inflammatory signaling pathways, including the nuclear factor kappa-light-chain-enhancer of activated B cells (NF-κB) (59–61), the mitogen-activated protein kinase (MAPK) (61, 62), and the signal transducer and activator of transcription-3 (STAT3) (63, 64), leading to the production of pro-inflammatory cytokines (e.g., IL-1β, IL-1Ra, IL-6, CXCL8, IL-10, and TNF-α) and chemokines (e.g., C-C motif chemokine ligand [CCL]2, CCL3, CCL18, and CCL20) (59, 60, 62–67). As such, eNAMPT can be viewed either as a pro-inflammatory cytokine or as an alarmin, in other words, a Damage-Associated Molecular Pattern (DAMP). In addition, eNAMPT can function directly as a chemokine to induce the recruitment of monocytes and B cells *in vitro*, as well as that of neutrophils and monocytes *in vivo* (32, 66). eNAMPT was able not only to induce immune cell chemotaxis but also to promote their proliferation and differentiation (63, 68). Studies have shown eNAMPT capable of inducing context-dependent monocyte differentiation into either classically activated or alternatively activated macrophages (69) (i.e., M1-type (31, 32, 70), or M2-type (63, 71), respectively). For example, M2 polarization was observed with monocytes from leukemic patients (63), whereas M1 polarization was reported with monocytes from healthy donors (31).

In general, eNAMPT appears as a modulator of innate immune pro-inflammatory programs, particularly in monocytes and macrophages. For instance, eNAMPT supported the survival of mouse macrophages by suppressing the ER-stress-induced apoptosis of these cells through the activation of STAT3 (64). eNAMPT also induced the expression of co-stimulatory molecules in human monocytes, such as CD40, CD54, and CD80 (66). As a result, eNAMPT was able to promote monocyte effector functions, in particular, phagocytosis (66). The pro-inflammatory functions of eNAMPT are not limited to immune cells. eNAMPT is also involved in vascular inflammation by increasing the expression of endothelial cell adhesion molecules (intercellular adhesion molecule 1 [ICAM-1] and vascular cell adhesion molecule 1 [VCAM-1]) in endothelial cells, which boost the adhesion of monocytes (72, 73). Another study reported that eNAMPT promotes NLR family pyrin domain containing 3 (NLRP3) inflammasome complex activation and the subsequent release of pro-inflammatory IL-1β from endothelial cells (74). Neither the enzymatic activity (31, 32, 63, 64, 75) nor dimerization (64) were required for these cytokine/DAMP activities of eNAMPT.

### 3.4 eNAMPT as a pro-angiogenic factor

In addition to its pro-inflammatory effects, eNAMPT was shown to play a substantial role in angiogenesis. For instance, eNAMPT induced the proliferation, migration, and capillary-tube-forming activity of human umbilical vein endothelial cells *in vitro* (76–78). The pro-angiogenic function of eNAMPT was also confirmed *in vivo*, in angiogenesis models (76). *In vitro*, eNAMPT was able to induce the production and secretion of vascular endothelial growth factor (VEGF), as well as VEGF receptor 2 expression in endothelial and amniotic epithelial cells via the activation of MAPK and phosphatidylinositol-3 kinase/protein kinase B/mammalian target of rapamycin (PI3K/AKT/mTOR) signaling pathways (77–81). Yet other studies have suggested that eNAMPT mediates angiogenesis by eliciting IL-6, CXCL8, fibroblast growth factor 2, and monocyte chemoattractant protein-1 release from endothelial cells (82–86). Finally, eNAMPT was also shown to participate indirectly in extracellular matrix remodeling, a process creating a microenvironment favoring angiogenesis, via the upregulation of proteases involved in vascular basal membrane degradation (MMPs; gelatinases: MMP-2 and -9 and collagenases: MMP-1 and -8) (61, 77, 87–89).

## 4 eNAMPT cell surface receptors

The rapid activation of specific intracellular pathways, which occurs within minutes of exposure to eNAMPT, suggests that this protein exerts its functions via binding to one or more cell surface receptors (2, 63). Over the last few years, several putative eNAMPT cell surface receptors have been identified. Two of these, in particular, are supported by solid experimental evidence: The first one is the C-C chemokine receptor type 5 (CCR5). In 2012, Van den Bergh et al. demonstrated the direct binding of eNAMPT to CCR5, with an affinity (Kd) in the nM range (90). Later, Torretta et al. found that eNAMPT has a similar structural conformation to CCL7, a known CCR5 ligand and proposed that eNAMPT, like CCL7, acts as an antagonist of CCR5 (91). Eventually, Ratnayake and colleagues confirmed the interaction between eNAMPT and CCR5 in an ELISA binding assay (92, 93).

More recently, studies have focused on the interaction between eNAMPT and Toll-like receptor 4 (TLR4). In 2015, Camp et al. revealed that eNAMPT activates the NF-κB pathway by interacting with TLR4 (94). Consistent with this, another study demonstrated that *TLR4* gene silencing in macrophages resulted in a significant reduction in eNAMPT-mediated NF-κB activation (59). Interestingly, eNAMPT has also been suggested to promote vascular dysfunction in mice through a TLR4-mediated pathway. In particular, the authors showed that a specific TLR4 inhibitor, CLI-095, prevented eNAMPT-mediated impairment of the endothelial cell responses to acetylcholine (74). Gasparrini et al. characterized the direct binding of eNAMPT to TLR4, unveiling a relatively high affinity value (KD of 18 nM (65)). Using site-directed mutagenesis, the authors identified two regions in the N-terminal part of eNAMPT that are involved in TLR4 binding (β1-β2 loop: 41–52 aa; and α1-α2 loop: 68–77 aa). The latter findings were recently confirmed by Kim et al. (95), who showed that the 57–65 aa region of eNAMPT interacts with the leucine-rich repeats (LRR) domain of TLR4. A putative TLR4-binding site in the C-terminal region of eNAMPT (445–457 aa) has also been identified (96).

Nonetheless, the question of biologically relevant eNAMPT receptors remains open-ended. For instance, Colombo et al. recently demonstrated that eNAMPT promoted the expression of inflammatory M1-related genes in macrophages (e.g*., Il6, Il1b, Cox2*, and *Tnf*), independently of TLR4 (32). They also reported that a competitive molecular antagonist of CCR5 (maraviroc) did not alter eNAMPT-induced activation of M1 macrophages (32). Furthermore, another group used the Retrogenix cell microarray platform to screen for potential eNAMPT protein-protein interactions with over 2,500 known human receptors (97). Using this approach, they identified multiple candidates, distinct from CCR5 and TLR4, suggesting that eNAMPT might interact rather pleiotropically with diverse receptors exposed at the cell surface (97).

## 5 eNAMPT in disease

**Figure 2:**
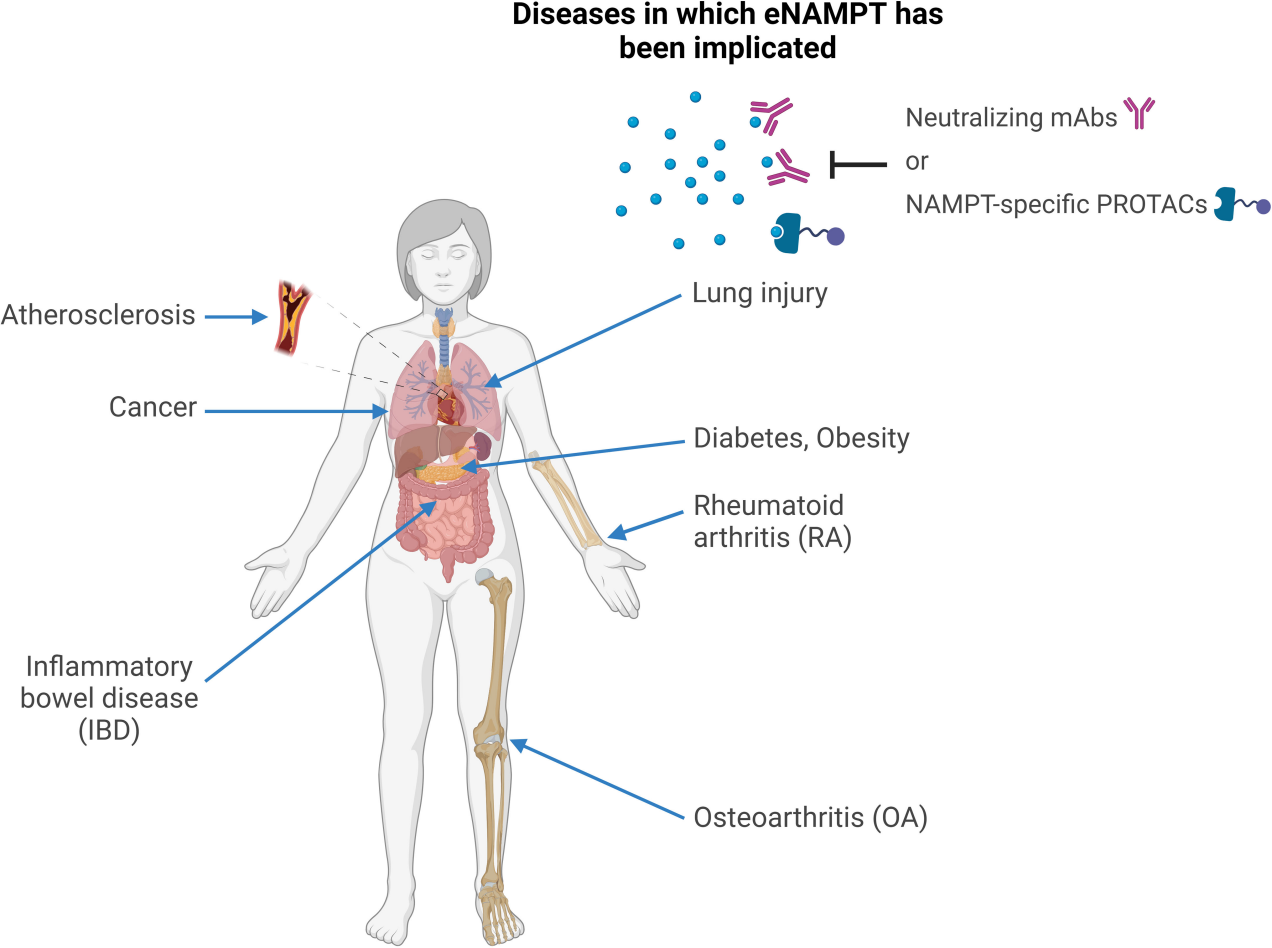
eNAMPT and human disease. Schematic representation of the human conditions in which eNAMPT was proposed to play a role. In all of these conditions, elevated eNAMPT levels were observed in patients’ sera and other bodily fluids, such as synovial fluid in rheumatoid arthritis or bronchoalveolar lavage in lung injury. In addition, preclinical studies pinpointed the mechanisms through which eNAMPT is likely to contribute to the pathophysiology of these conditions, mostly via pleiotropic effects on metabolism, inflammation, and immunity as a DAMP/adipocytokine/pro-angiogenic factor. For at least some of these diseases, neutralizing or reducing eNAMPT levels by monoclonal antibodies or by “molecular glues” (e.g., PROTACs), respectively, was shown to have therapeutic activity in preclinical models.

Elevated eNAMPT levels are characteristic of numerous human metabolic and inflammatory disorders. Accumulated clinical and preclinical research data suggests eNAMPT could be involved in the pathogenesis of these conditions (represented in **Figure 2**) and/or serve as a disease biomarker.

### 5.1 Metabolic and cardiovascular diseases

Over the past few years, multiple clinical studies have reported increased circulating eNAMPT concentrations in patients diagnosed with obesity, diabetes, or atherosclerosis, suggesting a role for eNAMPT as an universal biomarker for metabolic and cardiovascular diseases (19). Notably, numerous *in vitro* and *in vivo* studies suggest elevated eNAMPT levels might be a causative factor in these conditions, directly contributing to their pathophysiology.

#### 5.1.1 Obesity

##### 5.1.1.1 Preclinical studies

Similar to many other adipocytokines, eNAMPT secretion from adipose tissues is altered in obesity. One experimental study reported that plasma eNAMPT levels are significantly higher in obese, HFD-fed, mice than in controls (98). Whether eNAMPT contributes directly to the mechanisms underlying obesity remains still unclear. In any case, several studies have demonstrated that eNAMPT plays a role in the development of obesity-associated pathologies, including glomerular damage, endothelial dysfunction, and adipose tissue fibrosis (99). In particular, eNAMPT was shown to induce NLRP3 inflammasome, which is involved in the chronic, low-grade inflammation which is characteristic of obesity (74, 98, 100–102). Activation of the NLRP3 complex by eNAMPT was shown to promote glomerular injury, as evidenced by the increased expression of the injury factor desmin in podocytes (101). Moreover, Chen et al. (100) reported that eNAMPT-mediated NLRP3 inflammasome activation provoked the disassembly of junction proteins (zonula occludens [ZO]-1, ZO-2, occludin, and VE-cadherin) in mouse vascular endothelial cells, suggesting that eNAMPT promotes endothelial dysfunction. Finally, eNAMPT has been shown to enhance the expression of ECM proteins (e.g., collagen type C and osteopontin) and MMPs (MMP-2 and -9) in 3T3-L1 pre-adipocytes, leading to ECM accumulation and remodeling, and consequently, to adipose tissue fibrosis (87).

##### 5.1.1.2 Clinical studies

Mounting evidence suggests that eNAMPT could be an attractive complementary biomarker in obesity, in particular of the chronic low-grade inflammation that is associated with this condition. Numerous clinical studies have reported significantly higher circulating eNAMPT levels in obese patients than in lean controls [reviewed by Carbone et al. (3) and summarized in **Table 1** (87, 103–106, 117–120)]. Correlations between eNAMPT levels and unfavorable metabolic profiles, including high waist circumference and waist-to-hip ratio, as well as elevated levels of triglycerides, have also been observed (103, 118, 120). eNAMPT levels were also often positively correlated with body mass index (BMI) (87, 103, 104), although not in all studies (119, 121). These discrepancies could be explained by differences in the type of study, the type of population recruited, the number of cases per cohort, and the methods of sample collection and measurement (122). Consistent with the association between elevated eNAMPT levels and adiposity, several studies have reported a reduction in circulating eNAMPT levels after weight loss, and exercise has been shown to decrease eNAMPT levels in overweight/obese patients (123–125). Namely, Friebe et al. observed that serum eNAMPT concentrations became significantly reduced in obese subjects after bariatric surgery, and that this reduction correlated with a decline in white blood cell numbers (117). Another group demonstrated that in human visceral WAT, eNAMPT is mainly released by macrophages which accumulate with BMI increase (126, 127). Increased levels of circulating eNAMPT in obese patients are correlated with an inflammatory signature (e.g., IL-6, TNF-α, and C-reactive protein [CRP]) and endothelial markers (e.g., VCAM-1, ICAM-1, angiotensin-2 [ang-2], and E-selectin) (104, 120, 128). In addition, eNAMPT levels were also positively associated with endotrophin (r = 0.619, P < 0.001), a marker of increased fibrosis and metabolic abnormalities, in obese patients (87). Last but not least, plasma eNAMPT concentrations were shown to predict visceral adipose tissue accumulation in obese children (119). All this suggests a great potential for eNAMPT as a biomarker of obesity and chronic, low-grade inflammation. However, is it important to emphasise here that eNAMPT levels do not always correlate with metabolic and inflammatory disease. For example, no association was found between circulating eNAMPT levels and the severity of non-alcoholic fatty liver disease (manifested as fibrosis and non-alcoholic steatohepatitis) (129).

**Table 1:** eNAMPT as a potential biomarker in metabolic diseases.

Author	Year	Sample size	Results
Obesity			
**Ezzati-Mobaser et al. (** 87)	2020	30 obese children 29 controls	Significantly higher plasma eNAMPT levels were found in obese patients than in control subjects (P = 0.005). Positive correlations between eNAMPT and BMI z-score (r = 0.356, P = 0.006) and eNAMPT and endotrophin (r = 0.619, P < 0.001) were observed.
**Alnowihi et al. (** 103)	2020	35 obese women 15 overweight women 33 controls	The obese group had higher serum eNAMPT levels than the overweight and lean groups (P < 0.001). eNAMPT levels were positively correlated with waist and hip circumferences (r = 0.4, P ≤ 0.001), BMI (r = 0.5, P ≤ 0.001), DBP (r = 0.2, P = 0.02), SBP (r = 0.2, P = 0.02), insulin (r = 0.3, P ≤ 0.01), HOMA-IR (r = 0.2, P = 0.02), and LDL-C (r = 0.3, P ≤ 0.01) and negatively correlated with HDL-C (r = −0.2, P ≤ 0.01), E2 (r = −0.2, P = 0.04), and SHBG (r = −0.26, P ≤ 0.01) values.
**Yin et al. (** 104)	2019	160 obese children 84 controls	Serum eNAMPT levels were higher in obese children than in lean children (P < 0.01). Similar plasma eNAMPT concentrations were found in the insulin-resistant and non-insulin-resistant groups of obese children (P > 0.05). Serum eNAMPT levels correlated with SDS-BMI (β(r) = 0.601, P = 0.005), BMI (β(r) = 0.523, P = 0.008), hsCRP (β(r) = 0.633, P = 0.000), IL-6 (β(r) = 0.736, P = 0.000), TNF-α (β(r) = 0.733, P = 0.000), VCAM-1 (β(r) = 0.726, P = 0.000), ICAM-1 (β(r) = 0.953, P = 0.000), Ang-2 (β(r) = 0.933, P = 0.000), and E-selectin (β(r) = 0.896, P = 0.000) levels in obese children.
**Ahmadpour et al. (** 105)	2018	35 obese children 35 controls	Serum eNAMPT levels were significantly higher in obese children than in healthy controls (P < 0.001). A negative correlation between serum eNAMPT and plasma miR-149 levels was found (r = −0.302, P = 0.001).
**Nourbakhsh et al. (** 106)	2015	42 obese children 31 controls	Obese children had significantly higher plasma eNAMPT concentrations than lean controls (P < 0.001). In obese subjects, eNAMPT correlated positively with FPG (r = 0.34, P < 0.05), insulin (r = 0.58, P < 0.01), and HOMA-IR (r = 0.57, P < 0.01).
Diabetes			
**Mir et al. (** 107)	2022	87 T2DM 85 controls	Male T2DM patients had significantly higher serum eNAMPT levels than controls (P < 0.01).
**Mostafa et al. (** 108)	2021	60 T2DM 60 controls	Circulating levels of eNAMPT were significantly higher in T2DM patients than in control individuals (P < 0.001). Correlations of eNAMPT with FBG (r = 0.621, P < 0.01), insulin (r = 0.416, P < 0.01), HOMA-IR (r = 0.518, P < 0.01), HbA_1c_ (r = 0.388, P < 0.01), IL-34 (r = 0.312, P < 0.01), IRAPe (r = −0.380, P < 0.01), and irisin (r = −0.494, P < 0.01) were observed.
**Sayers et al. (** 109)	2020	27 obese T2DM 15 obese IFG 13 controls	Obese patients with T2DM had significantly higher levels of serum eNAMPT than obese individuals with impaired fasting glucose (P < 0.05) and non-diabetic obese controls (P < 0.05). Furthermore, eNAMPT levels correlated with HbA_1c_ levels (r^2^ = 0.379, P < 0.01). In non-diabetic individuals, 4.2% of NAMPT was monomeric, versus 29% in T2DM patients.
**Bawah et al. (** 110)	2019	70 GDM pregnant women 70 controls	During the first trimester, higher serum eNAMPT levels were found in women who subsequently developed GDM (P < 0.0001). Increased levels of eNAMPT were associated with GDM [OR = 1.342 (CI = 1.185–1.518), P < 0.0001]. Furthermore, a ROC analysis showed that eNAMPT levels can predict GDM (sensitivity = 87.1%, specificity = 70%, AUC = 0.799).
**Hetta et al. (** 111)	2018	80 T2DM 40 controls	T2DM patients had higher levels of serum eNAMPT than controls (P = 0.001). In the T2DM group, a positive correlation was found between eNAMPT levels and IL-6 (r = 0.47, P < 0.0001), TNF-α (r = 0.62, P < 0.0001), CRP (r = 0.40, P < 0.002), WC (r = 0.38, P = 0.001), BMI (r = 0.40, P = 0.008), and IR (r = 0.48, P = 0.001).
Atherosclerosis			
**Zheng et al. (** 112)	2019	56 T2DM patients with plaques 41 T2DM patients without plaques	Patients with atherosclerotic plaques had higher serum eNAMPT levels than patients without plaques (male subgroup: P = 0.005; female subgroup: P = 0.008). Positive correlations were observed between serum eNAMPT levels and WC (r = 0.226, P = 0.029), waist-hip ratio (r = 0.221, P = 0.032), TG (r = 0.222, P = 0.030) and number of plaques (r = 0.275, P = 0.009). Furthermore, elevated concentrations of serum eNAMPT was shown to be an independent predictor of the presence of atherosclerotic plaques (OR = 3.315 [CI = 1.496–7.349], P = 0.003).
**Auguet et al. (** 113)	2016	18 patients with unstable carotid atherosclerotic plaques 13 coronary patients with non-atherosclerotic mammary arteries 16 controls	Higher levels of eNAMPT were found in the secretome of patients with atherosclerotic plaques than in those with non-atherosclerotic mammary arteries (P = 0.021). Serum eNAMPT levels were increased in patients with unstable carotid atherosclerotic plaques (P = 0.037) and non-atherosclerotic mammary arteries (P = 0.001) compared with those in healthy controls.
**Kong et al. (** 114)	2014	39 acute ischemic cerebral infarction patients with atherosclerosis 21 patients without atherosclerosis 35 healthy controls	Mean serum eNAMPT levels were significantly higher in patients with atherosclerosis than in those without (P < 0.001). Positive correlations of eNAMPT with TG (P = 0.027) and glucose (P = 0.004) were shown. No correlations were observed between eNAMPT levels and BMI, TC, HDL-C, or LDL-C (P > 0.05). A logistic regression analysis revealed that eNAMPT levels were a risk factor for atherosclerosis (χ2 = 8.515, OR = 37.797, P = 0.004).
**Kadoglou et al. (** 115)	2012	74 patients with carotid atherosclerosis 38 controls	Patients with carotid atherosclerosis had significantly higher serum eNAMPT levels than controls (P < 0.001). eNAMPT negatively correlated with GSM score (r = −0.366, P = 0.022). eNAMPT levels were also significantly correlated with hsCRP (β = 0.499, P < 0.001) and BMI (β = 0.112, P = 0.038).
**Zhong et al. (** 116)	2008	40 MetS patients with carotid plaques 99 MetS patients without carotid plaques 105 controls	Serum eNAMPT levels were significantly higher in MetS patients with carotid plaques than in patients without (P < 0.001) or in controls (P < 0.001). LogNAMPT concentration in MetS patients correlated with max-IMT (β = 0.293, P = 0.001) and LDL-c (β = 0.219, P = 0.013) in the multiple regression analysis.

Previously reviewed by Carbone et al. (3) and Garten et al. (1). Ang-2, angiotensin-2; AUC, area under the receiver operating characteristic curve; BMI, body mass index; CI, confidence interval; CRP, C-reactive protein; DBP, diastolic blood pressure; E2, estradiol; eNAMPT, extracellular nicotinamide phosphoribosyltransferase; FBG, fasting blood glucose; FPG, fasting plasma glucose; GDM, gestational diabetes mellitus; GSM, gray scale median; HbA_1c_, hemoglobin A1C; HDL-C, high-density lipoprotein-cholesterol; hs, high-sensitivity; HOMA-IR, homeostatic model assessment of insulin resistance; ICAM-1, intercellular adhesion molecule-1; IFG, impaired fasting glucose; IL, interleukin; IMT, intima-media thickness; IR, insulin resistance; LDL-C, low-density lipoprotein-cholesterol; MetS, metabolic syndrome; OR, odds ratio; ROC, receiver operating characteristic; SBP, systolic blood pressure; SDS-BMI, BMI standard deviation score; SHBG, sex hormone-binding globulin; T2DM, type 2 diabetes mellitus; TC, total cholesterol; TG, triglycerides; TNF-α, tumor necrosis factor-α; VCAM-1, vascular cell adhesion molecule-1; WC, waist circumference.

#### 5.1.2 Diabetes

##### 5.1.2.1 Preclinical studies

The role of eNAMPT in the pathophysiology of diabetes is controversial. Multiple studies have reported a beneficial effect of eNAMPT as an ectoenzyme on pancreatic function. For instance, eNAMPT and its product, NMN, were shown to maintain β-cell homeostasis by modulating cellular NAD levels (18). What is more, impaired islet function of mice fed a fructose-rich diet was associated with reduced plasma eNAMPT levels, which could be reversed by NMN administration (57). In line with this, injection of NMN into aged β-cell-specific SIRT-1-overexpressing transgenic mice restored the beneficial effect of SIRT-1 on glucose tolerance, which was lost with mice ageing (56). Conversely, one study reported that prolonged eNAMPT administration induced a diabetic phenotype in mice (130); suggesting that abnormally elevated eNAMPT levels may exert a detrimental effect on β-cell activity. Thus, the bimodal effect of eNAMPT on diabetes may be concentration-dependent: physiological concentrations of eNAMPT may help maintain pancreatic function, whereas higher concentrations of eNAMPT may drive pathological mechanisms (109). The authors demonstrated that low, physiological levels of eNAMPT increased static and dynamic glucose-stimulated insulin secretion (GSIS) and intracellular cytosolic calcium levels in mouse islets, whereas higher levels of eNAMPT resulted in islet inflammation and β-cell failure (109). These findings suggest that circulating blood levels of eNAMPT and its enzymatic activity may differentially affect diabetes pathophysiology.

##### 5.1.2.2 Clinical studies

Multiple clinical studies have investigated the correlation between eNAMPT levels and the different types of diabetes. Increased eNAMPT levels are detected in patients with type 1 diabetes mellitus (T1DM) (131, 132), type 2 DM (T2DM) (107–109, 111, 132–135), and gestational diabetes (110, 136), compared with non-diabetic individuals (detailed in **Table 1**). The increase in serum eNAMPT levels coincided with the severity of diabetes, and particularly with the progression of β-cell deterioration, a major feature of T2DM. This was further attested by the positive correlation between eNAMPT and the levels of hemoglobin A1c, an important marker of glycemic control, whose levels directly correlate with the concentration of sugar in the blood during the previous three months (109, 132). The clinical association between eNAMPT and diabetes was independent of BMI (133, 134). No significant differences in circulating eNAMPT concentrations were found between pre-diabetic subjects and healthy controls (135). In addition, the levels of eNAMPT in newly diagnosed T2DM patients were similar to those of non-diabetic individuals, whereas eNAMPT levels of patients with chronic T2DM were higher (132). This suggests that elevated eNAMPT may play a role in the later stages of the disease, probably contributing to the maintenance of lingering inflammation. The link between eNAMPT and insulin resistance, another major characteristic of T2DM pathophysiology, was also extensively explored. Some studies (19, 108, 111, 137), but not others (138, 139), showed an association between eNAMPT levels and homeostasis model assessment of insulin resistance (HOMA-IR – in other words, a correlation between elevated eNAMPT and insulin resistance). Higher serum eNAMPT levels were also found in insulin-resistant children compared to insulin-sensitive children (117). Likewise, elevated eNAMPT levels were shown to predict gestational diabetes with high sensitivity and specificity (110). In conclusion, eNAMPT levels could still be a promising biomarker for monitoring diabetes progression, at least in specific medical conditions.

#### 5.1.3 Atherosclerosis

##### 5.1.3.1 Preclinical studies

Along with serum lipid levels, plasma eNAMPT levels are significantly elevated in *ApoE* knockout atherosclerotic mice (140). eNAMPT is also highly expressed in human symptomatic atherosclerotic plaques, localized to areas where foam cells are abundant (67, 141). Studies have shown that pro-atherogenic stimuli (e.g., oxidized low-density lipoprotein, hypoxia, and TNF-α) increase eNAMPT expression in THP-1 monocytes (67, 142) and promote eNAMPT release from RAW264.7 macrophage-like cells (140). In turn, elevated eNAMPT triggers cholesterol uptake and accumulation in macrophages (140) and induces the secretion of pro-inflammatory cytokines in peripheral blood mononuclear cells from patients with unstable angina (67). Thus, through its pro-inflammatory actions, eNAMPT may participate in foam cell formation and fatty streak development during atherogenesis. eNAMPT could also contribute to atherosclerosis development by promoting endothelial dysfunction. In this context, eNAMPT has been shown to alter the migration and adhesion of endothelial progenitor cells (EPCs, key cells in the regeneration of impaired blood vessels), and to induce their apoptosis (143, 144). What is more, eNAMPT may also directly impair microvascular endothelium-dependent vasorelaxation by reducing the endothelial response to vasodilators such as acetylcholine (74, 145), negatively affect vessel wall integrity and compound plaque stability by promoting the expression of MMPs in monocytes, macrophages (67, 88) and endothelial cells (77). Finally, eNAMPT could induce human vascular smooth muscle cell proliferation (42) and upregulate their nitric oxide synthase levels (146), thereby promoting vascular inflammation.

##### 5.1.3.2 Clinical studies

Consistent with the experimental findings, elevated circulating eNAMPT levels were observed in patients with atherosclerotic plaques [see **Table 1** (112–116, 147)]. Increased plasma eNAMPT concentrations were also measured after mechanical induction of plaque rupture (67) or in patients diagnosed with acute myocardial infarction (148), suggesting a relationship between eNAMPT levels and plaque instability. In line with that, a negative correlation between eNAMPT concentration and the gray-scale median score, which quantifies plaque vulnerability, in atherosclerotic patients, was found (115). Circulating eNAMPT concentrations are correlated with the extent of atherosclerosis, as measured by intima media thickness (116, 147) and with endothelial dysfunction, based on flow-mediated vasodilatation (149). Elevated circulating eNAMPT levels were proposed to be a risk factor for the development of atherosclerotic plaques (112, 114).

### 5.2 Osteoarthritis

#### 5.2.1 Preclinical studies

Several studies have suggested that eNAMPT promotes tissue degradation in OA. For instance, Cheleschi et al. reported that eNAMPT contributes to cartilage turnover by promoting the apoptosis of OA chondrocytes and OA synovial fibroblasts (OASFs) (150, 151). eNAMPT can also contribute to joint degeneration during OA by affecting oxidative stress, in particular mitochondrial superoxide anion production as well as the expression of antioxidant enzymes (e.g., superoxide dismutase, catalase [CAT], and nuclear factor erythroid 2-related factor 2) (150–153). In addition, eNAMPT may exacerbate OA by promoting the expression of a variety of proteases: Studies show that eNAMPT stimulates the release of sulphated glycosaminoglycans from cartilage and meniscus explants, which is suggestive of elevated aggrecanase activity and proteoglycan loss (97, 154, 155). Gosset et al. reported that eNAMPT reduces aggrecan mRNA and the levels of high molecular weight aggregated proteoglycan in immature mouse articular chondrocytes and induces the expression of ADAMTS-4 and ADAMTS-5 aggrecanases (156). eNAMPT was also shown to upregulate the expression of MMPs (collagenases: MMP-1, -8, -13; gelatinases: MMP-2, -9; stromelysins: MMP-3, -10; matrilysin: MMP-7) in OA chondrocytes and OASFs (97, 150, 151, 156). In human and mouse OA chondrocytes, eNAMPT promoted the synthesis of prostaglandin E2, a well-known cartilage catabolic factor (156, 157). Indeed, eNAMPT-induced cartilage degradation seems to result, in large part, from its DAMP/pro-inflammatory activities, as eNAMPT was shown to activate NF-κB and MAPK signaling pathways in OASFs, leading to the downstream expression of pro-inflammatory cytokines and the activation of inflammatory processes (40, 97, 151, 158). On the other hand, eNAMPT may not only stimulate the activity of catabolic processes but could also directly alter the expression of cartilage structural proteins, thereby impairing cartilage production. For instance, eNAMPT was shown to reduce the expression of collagen type II and type X in OA chondrocytes and OASFs (151, 159). By the same token, another study demonstrated that eNAMPT prevents the insulin-like-growth-factor-1-mediated production of collagen type II and proteoglycan (160). Finally, another effect of eNAMPT was to increase VEGF production by human OASFs, which in turn induces EPC angiogenesis, a process involved in structural damage and pain during OA (161, 162). Also, Pecchi et al. revealed that eNAMPT induces nerve growth factor expression and release by chondrocytes, supporting the idea of a possible involvement of this DAMP/cytokine in OA-associated pain (163).

#### 5.2.2 Clinical studies

Several studies have reported significantly elevated eNAMPT levels in the serum or synovial fluid of OA patients (see **Table 2** (161, 164–167),). eNAMPT found in OA joints was shown to be released from many tissue components, including the synovium, the subchondral bone and cartilage, and the infrapatellar fat pad (40, 167). In line with preclinical evidence, synovial fluid eNAMPT levels positively correlated with OA severity and with biomarkers of cartilage degradation (166). eNAMPT was also positively associated with VEGF levels and with pain score (161, 179). As a corollary, experimental and clinical studies strongly suggest eNAMPT may serve as a complementary biomarker and/or a potential drug target in OA (180).

**Table 2:** eNAMPT as a potential biomarker in autoimmune and inflammatory diseases.

Author	Year	Sample size	Results
Osteoarthritis			
**Askari et al. (** 164)	2020	150 OA patients 300 controls	Higher serum eNAMPT levels were found in OA patients than in controls (P < 0.001). eNAMPT levels correlated positively with OA (β = 24.71, P < 0.001).
**Tsai et al. (** 161)	2020	30 OA patients 30 controls	OA patients had significantly increased serum eNAMPT concentrations than controls (P < 0.0001). A positive correlation was observed between serum eNAMPT and VEGF levels (r = 0.668, P < 0.0001).
**Fioravanti et al. (** 165)	2018	47 erosive hand OA patients 21 controls	Higher serum eNAMPT levels were found in patients with erosive hand OA than in controls (P < 0.0005).
**Duan et al. (** 166)	2012	30 women with OA 12 controls	Higher synovial, but not plasma, eNAMPT levels were found in OA patients compared with healthy individuals (P < 0.001). Patients with a radiological KL grade 4 presented significantly higher SF eNAMPT levels compared to patients with radiological KL grade 3 (P = 0.001). Furthermore, positive correlations were found between SF eNAMPT levels and SF CTX-II (r = 0.497, P = 0.005) and SF aggrecans fragments, AGG1 (r = 0.451, P = 0.012), and AGG2 (r = 0.434, P = 0.017).
**Chen et al. (** 167)	2010	23 OA patients 17 controls	OA patients had significantly increased serum eNAMPT levels than controls (P < 0.05). Notably, in OA patients, the SF eNAMPT levels were higher than the paired serum eNAMPT levels (P = 0.004). Serum eNAMPT concentrations correlated positively with SF eNAMPT levels (r = 0.79, P < 0.001).
Rheumatoid arthritis			
**Cheleschi et al. (** 168)	2022	50 RA patients 50 controls	Increased serum eNAMPT levels were found in RA patients compared with healthy controls (P < 0.001).
**Ali et al. (** 169)	2020	60 RA patients 30 controls	Serum eNAMPT concentrations were significantly more elevated in RA patients compared with controls (P = 0.013). In this study, eNAMPT levels correlated with those of chemerin (r = 0.328, P ≤ 0.01) and with the chemerin/eNAMPT ratio (r = −0.599, P ≤ 0.01).
**Sglunda et al. (** 170)	2014	40 RA patients 30 controls	Serum eNAMPT levels were significantly higher in RA patients than in healthy controls (P = 0.034). eNAMPT was positively correlated with DAS28 (r = 0.383, P = 0.015) and CRP levels (r = 0.456, P = 0.003) and negatively correlated with anti-CCP levels (r = −0.400, P = 0.011). Of note, serum eNAMPT concentrations decreased significantly after 3 months of treatment with nonsteroidal antirheumatic drugs, compared with baseline values (P < 0.0001). This reduction in eNAMPT levels was associated with a decrease in DAS28 (r = 0.378, P = 0.018) and CRP (r = 0.386, P = 0.015) and predicted an amelioration in DAS28 after 12 months of treatment (P = 0.031, R^2^ = 10.1%). Finally, in RA patients, the reduction in eNAMPT levels significantly predicted a rise in the amount of total cholesterol (P = 0.015).
**El-Hini et al. (** 171)	2013	40 RA patients 40 controls	Elevated serum eNAMPT levels were found in RA patients compared with healthy controls (P = 0.000). In addition, eNAMPT concentrations positively correlated with insulin (r = 0.303, P = 0.05), HOMA-IR (r = 0.346, P = 0.029), HOMA-B (r = 0.434, P = 0.005), cholesterol (r = 0.501, P = 0.001), TG (r = 0.471, P = 0.002), LDL-C (r = 0.319, P = 0.045), and the disease activity score (DAS moderate: r = 0.329, P = 0.038; DAS severe > 5: r = 0.528, P = 0.003). A negative correlation was also found between eNAMPT levels and HDL-C (r = −0.398, P = 0.011) and adiponectin (r = −0.686, P = 0.000).
**Šenolt et al. (** 172)	2011	29 RA patients 33 controls	Serum eNAMPT levels were significantly increased in RA patients than in healthy controls (P = 0.026). Of note, eNAMPT levels of RA patients at week 16 of rituximab treatment were close to those of controls (P = 0.086). In addition, serum eNAMPT levels correlated positively with the total number of B cells (rs = 0.417, P = 0.025). However, eNAMPT levels did not correlate with DAS28 (rs = −0.021, P = 0.913), with CRP (rs = 0.091, P = 0.640), or with IgM-RF levels (rs = −0.276, P = 0.147).
Inflammatory bowel disease			
**Colombo et al. (** 30)	M2023	180 IBD patients (128 CD, 52 UC) 18 controls	Higher serum eNAMPT levels were found in IBD patients compared with controls (P < 0.0001). No differences in eNAMPT concentrations were observed between UC and CD patients (P > 0.05). Serum eNAMPT concentrations correlated positively with hsCRP (r = 0.62, P = 0.0001), severity score (r = 0.40, P = 0.0001), BMI (r = 0.56, P = 0.001) and IL-6 (r = 0.36, P = 0.001) and negatively with IL-10 (r = -0.55, P = 0.001). eNAMPT levels were significantly lower in patients responsive to adalimumab treatment than in non-responsive patients (P < 0.001). Furthermore, an ROC analysis showed that eNAMPT levels could be used to discriminate between responsive and non-responsive patients (AUC = 0.71, CI = 0.56–0.86).
**Saadoun et al. (** 173)	2021	85 IBD patients (56 UC, 29 CD) 30 controls	Increased serum eNAMPT levels were found in IBD patients compared with healthy controls (P < 0.001). In UC patients, eNAMPT concentrations correlated with BMI (r = 0.31, P = 0.02), CRP (r = 0.58, P < 0.001), ESR (r = 0.58, P < 0.001), TLC (r = 0.28, P = 0.04), FC (r = 0.69, P < 0.001), and serum albumin (r = −0.54, P < 0.001). In CD patients, eNAMPT levels correlated positively with ESR (r = 0.44, P = 0.02), CRP (r = 0.48, P = 0.01), and FC (r = 0.64, P < 0.001). A ROC analysis demonstrated that eNAMPT levels could be used to accurately detect active UC (sensitivity = 92.9%, specificity = 86.7%, AUC = 0.911).
**Neubauer et al. (** 174)	2019	240 IBD patients (127 UC, 113 CD) 20 IBS-controls 40 controls	Higher serum eNAMPT levels were found in IBD patients than in IBS patients and healthy controls (P = 0.018). In addition, patients with active UC had significantly higher serum eNAMPT levels than patients with inactive UC (P = 0.001); no differences were found between patients with active and inactive CD. In UC patients, eNAMPT levels correlated positively with the Rachmilewitz index (ρ = 0.28, P = 0.002) and the Mayo endoscopic score (ρ = 0.47, P < 0.0001). eNAMPT levels correlated negatively with iron (UC: r = −0.30, P = 0.006; CD : r = −0.34, P = 0.003), Tf (UC: r = −0.34, P = 0.001; CD: r= −0.28, P = 0.018), and Alb (UC: r = −0.28, P = 0.006; CD: r= −0.35, P = 0.001) and positively with PLT (UC: r = 0.20, P = 0.030), WBC (UC: r = 0.18, P = 0.044; CD: r = 0.27, P = 0.009), ESR (UC: r = 0.41, P < 0.001), and hsCRP (UC: r = 0.37, P = 0.002; CD: r = 0.35, P = 0.013). Serum eNAMPT levels were also positively correlated with NAMPT expression in leucocytes in 16 IBD patients (r = 0.77, P < 0.001).
**Dogan et al. (** 175)	2016	31 UC patients 29 controls	Serum eNAMPT levels were significantly higher in patients with active UC than in controls (P < 0.05). In addition, eNAMPT levels were significantly higher in the active disease period compared with the remission period (P = 0.01).
**Waluga et al. (** 176)	2014	40 IBD patients (16 UC, 24 CD) 16 controls	Higher serum eNAMPT levels were found in IBD patients compared with healthy controls (P < 0.05) Notably, corticosteroids and/or azathioprine treatment significantly reduced eNAMPT concentration (P < 0.05) in CD patients, although these levels remained higher than those in healthy controls (P < 0.05).
**ALI / ARDS** **Bime et al. (** 177)	2022	Study cohort 1 (248 ARDS patients 70 controls) Study cohort 2 (100 ARDS patients 78 controls)	In patients with ARDS, plasma eNAMPT concentrations were significantly more elevated than in healthy controls (P < 0.01). Furthermore, eNAMPT levels could be used to discriminate between ARDS patients and healthy individuals, as shown by the ROC analysis (Cohort 1: AUC = 0.86, 95% CI = 0.82–0.90, P < 0.001; Cohort 2: AUC = 0.85, 95% CI = 0.8–0.9, P < 0.0001).
**Lee et al. (** 178)	2019	110 ALI patients 32 controls	Serum eNAMPT levels were higher in ALI patients than in control individuals (P < 0.001). A negative correlation between eNAMPT levels and survival rate of ALI patients was found (P = 0.002). In addition, eNAMPT levels positively correlated with those of IL-6 (ρ = 0.584, P < 0.001), IL-8 (ρ = 0.313, P = 0.001), IL-10 (ρ = 0.319, P = 0.001), and MCP-1 (ρ = 0.242, P = 0.011).
**Ye et al. (** 27)	2005	3 ALI patients 3 controls	Higher serum and BAL eNAMPT levels were found in ALI patients compared with healthy controls (P < 0.01).

Alb, albumin; ALI, acute lung injury; ARDS, acute respiratory distress syndrome; AUC, area under the curve; BAL, bronchoalveolar lavage; BMI, body mass index; CCP, cyclic citrullinated peptide; CD, Crohn’s disease; CI, confidence interval; CRP, C-reactive protein; CTX-II, C-terminal telopeptide of collagen type II; DAS28, disease activity score-28; eNAMPT, extracellular nicotinamide phosphoribosyltransferase; ESR, erythrocyte sedimentation; FC, fecal calprotectin; HDL-C, high-density lipoprotein-cholesterol; hs, high-sensitivity; HOMA-B, homeostatic model assessment of β-cell function; HOMA-IR, homeostatic model assessment of insulin resistance; IBD, inflammatory bowel disease; IBS, irritable bowel syndrome; IgM, immunoglobulin M; IL, interleukin; KL, Kellgren-Lawrence; LDL-C, low-density lipoprotein-cholesterol; MCP-1, monocyte chemoattractant protein-1; OA, osteoarthritis; PLT, platelet count; RA, rheumatoid arthritis; RF, rheumatoid factor; ROC, receiver operating characteristic; SF, synovial fluid; TLC, total leucocyte count; Tf, transferrin; TG, triglycerides; UC, ulcerative colitis; VEGF, vascular endothelial growth factor; WBC, white blood cells.

### 5.3 Rheumatoid arthritis (RA)

#### 5.3.1 Preclinical studies

As in OA, elevated eNAMPT levels may contribute to the pathogenesis of RA, an autoimmune disease which is also characterized by joint damage. However, as compared to OA, human RA tissues exhibit higher serum and synovial fluid concentrations of eNAMPT, probably reflecting a higher-grade inflammatory state of the latter (25, 181). Accordingly, eNAMPT levels were particularly high in the serum and in the paw tissues of collagen-induced arthritis mice, an experimental model of RA (182). In human RA synovial fibroblasts (RASFs), eNAMPT secretion is induced by pro-inflammatory cytokines and TLR ligands such as poly(I:C) and LPS, typically found in RA joints (25, 181). In turn, elevated eNAMPT levels induce the expression of pro-inflammatory cytokines (e.g., IL-6 and CXCL8), chemotactic signals (e.g., chemokines of the CXC and CC family) and matrix-degrading enzymes (e.g., MMP-1 and MMP-3) promoting a vicious circle of self-propagating inflammation and destruction of RA joints (25, 183–185). In addition, eNAMPT was also proposed to promote cartilage invasion by RASFs by increasing their adhesion to endothelial cells (186) and enhancing their motility and migration (184).

#### 5.3.2 Clinical studies

A growing body of research suggests that eNAMPT may be a useful biomarker for various autoimmune diseases characterized by chronic inflammation, in particular rheumatoid arthritis (RA) (187), inflammatory bowel disease (IBD) (188), and possibly also for psoriasis (189) and systemic lupus erythematosus (190).

As pointed out above, eNAMPT levels are high in RA (reviewed by Franco-Trepat et al. (187) and shown in **Table 2** [(168–172, 191–194)]. In RA patients, eNAMPT was strongly expressed in the synovial lining layer and at sites of RASF invasion in the cartilage (25, 184). The protein was also detected in lymphoid aggregates and in perivascular areas (25, 181, 184). eNAMPT levels positively correlate with inflammatory mediators like TNF-α, IL-6, and CRP and with immune cells counts, namely neutrophils and B cells, both in the serum and in synovial fluid of RA patients (25, 170, 172, 191, 192). Overall, eNAMPT levels increase with RA severity and duration (170, 171, 191, 193) and are particularly high in patients showing radiographic joint damage (191, 193). In turn, a significant decrease in circulating eNAMPT levels could be observed in some RA studies following treatment with conventional synthetic disease modifying antirheumatic drugs (csDMARDs) (170), TNF-α blockers (195), anti-CD20 antibody (172), or with a combination of methotrexate and anti-IL-6 therapy (196). What is more, a reduction in serum eNAMPT levels after three months of csDMARD treatment could predict an improvement of disease activity score, suggesting that eNAMPT may serve as a complementary prognostic biomarker in RA (170). That said, other RA studies do not support this concept, as no significant changes in circulating eNAMPT levels have been observed there, either following treatment with DMARDs, TNF-α blockers, or a combination of the two (197, 198).

### 5.4 Inflammatory bowel disease (IBD)

#### 5.4.1 Preclinical studies

While experimental studies of NAMPT role in IBD have mainly focused on the intracellular enzyme (199–201), Colombo et al. (188) recently reported that the administration of recombinant eNAMPT to mice with mild colitis exacerbated mucosal inflammation. They also observed an increased expression of TNF-α and IL-1β, and degradation of IκB-α in colonic tissues of eNAMPT-treated mice. Interestingly, an anti-NAMPT neutralizing monoclonal antibody was able to reduce the recruitment of pro-inflammatory monocytes and neutrophils and the activation of pathogenic Th1 and cytotoxic effector T cells in the colon in the dinitrobenzene sulfonic acid (DNBS)-induced model of colitis (188). The authors proposed that eNAMPT may fuel colonic inflammation by activating pro-inflammatory functions of myeloid cells and by triggering pathological Th1 and Th17-responses. Further studies are probably required to establish the role that eNAMPT plays in the pathogenesis of IBD, and particularly, in mucosal inflammation, a hallmark of this group of diseases.

#### 5.4.2 Clinical studies

A bulk of clinical studies in IBD reported elevated circulating eNAMPT levels in patients with ulcerative colitis (UC) and Crohn’s disease (CD) [**Table 2** (30, 173–176, 202–204)]. Interestingly, eNAMPT levels were found to be differently associated with disease activity in UC and CD patients. In CD patients, serum eNAMPT was elevated regardless of disease activity, whereas in UC patients, eNAMPT levels were significantly higher in patients with active disease as compared to inactive disease (66, 174, 175). Similar to the pattern observed in other inflammatory diseases, a positive correlation between the levels of eNAMPT and inflammation markers (e.g., IL-6, platelet count, erythrocyte sedimentation rate, or CRP) is apparent in IBD (173, 174). Intriguingly, Colombo et al. reported that after 14 weeks of therapy, IBD patients responding to anti-TNF-α treatment exhibited a strong decrease in circulating eNAMPT levels, while non-responders maintained elevated eNAMPT serum concentrations (188), suggesting that eNAMPT might be used as a predictive biomarker in IBD. Likewise, serum eNAMPT levels were shown to predict recurrence of active UC with a high sensitivity and specificity, underpinning the potential of eNAMPT as a diagnostic tool (173).

### 5.5 Lung injury

#### 5.5.1 Preclinical studies

Growing evidence implicates eNAMPT in the lung injury-associated inflammatory responses that are associated with various pulmonary conditions, such as acute lung injury (ALI), acute respiratory distress syndrome (ARDS; a severe form of ALI), and ventilator-induced lung injury (VILI). Neutralization of eNAMPT with an antibody was shown to attenuate inflammatory lung injury in preclinical mouse, rat, and porcine ARDS/VILI models (205–207) and serum eNAMPT was found to be increased in different ALI mouse models (178). As with most, if not all, diseases in which eNAMPT is thought to be implicated, eNAMPT’s effects appear to result from its pro-inflammatory DAMP activities. *In vivo* studies show that eNAMPT acts as a leucocyte chemoattractant increasing polymorphonuclear leucocyte (PMN) counts and chemokine levels in bronchoalveolar lavage (BAL) fluid (94, 208). Consistent with this, tracheal administration of eNAMPT was shown to augment BAL pro-inflammatory cytokine levels in mice (208). eNAMPT also exacerbated mechanical VILI features, as evidenced by alveolar wall thickening and neutrophil infiltration observed in a murine model (208). In addition, eNAMPT may also elicit lung injury by promoting endothelial cell barrier disruption: For example, Quijada et al. observed a decrease in transendothelial electrical resistance in human lung endothelial cells treated with eNAMPT, reflecting a loss of endothelial barrier integrity (206). In line with these findings, silencing of *NAMPT* in human pulmonary artery endothelial cells, attenuated thrombin-induced endothelial barrier dysfunction (209). Finally, eNAMPT was shown to further contribute to pulmonary permeability by dysregulating NF-κB, MAPK, and AKT-mTOR-Rictor signaling pathways in human lung endothelial cells (94, 205, 206).

#### 5.5.2 Clinical studies

Circulating levels of eNAMPT are increased in patients with ALI, ARDS [see **Table 2** (27, 177, 178)], and ARDS-predisposing conditions like sepsis or acute pancreatitis (177, 210), and seem to be correlated with survival in patients presenting with these conditions. For instance, higher eNAMPT levels were found in non-survivors of sepsis-induced ARDS than in survivors (211), and the survival rate of ALI patients was also shown to negatively correlate with serum eNAMPT (178). Understandably, eNAMPT now became one of a panel of six prognostic biomarkers for 28-day ARDS mortality (212). Supporting a link between eNAMPT and pulmonary inflammation, a positive correlation was found between elevated serum eNAMPT levels and IL-6, CXCL8, IL-10, and MCP-1 in ALI patients (178, 211). Importantly, circulating eNAMPT levels can discriminate between healthy individuals and patients with ARDS (177) or ARDS-predisposing pathologies (177, 210). Taken together, these findings indicate that eNAMPT may serve as a novel diagnostic/prognostic biomarker for pulmonary inflammatory conditions, and, possibly, as a tool for patient stratification in clinical trials.

### 5.6 Cancer

#### 5.6.1 Preclinical studies

Among all the pathologies that were investigated, elevated eNAMPT levels are probably the best documented in cancer. High levels of circulating eNAMPT are associated with various cancer types, ranging from solid tumors to hematological malignancies (213). eNAMPT, secreted both by the tumor (31, 34, 75) and by tumor-associated cells, is proposed to have a role in different hallmarks of cancer, promoting inflammation and cancer progression. To start with, eNAMPT could directly stimulate cancer cell proliferation *in vitro*, e.g., of breast cancer (41, 214–216), melanoma (217), hepatocellular carcinoma (218, 219), endometrial carcinoma (220), and prostate adenocarcinoma (221). eNAMPT is believed to exert its proliferative effects by activating various signaling pathways (c-Abl/STAT3, PI3K/AKT, MAPK/ERK, and Notch1/NF-κB) (214–216, 220) or by inducing the expression of cyclin D1 and cyclin-dependent kinase 2 (222). Moreover, eNAMPT was also shown to protect cancer cells from apoptosis by inhibiting survivin degradation (215) and from hydrogen-peroxide-induced DNA damage, presumably by increasing the activity of antioxidant enzymes (217). Some of these pro-tumorigenic effects of eNAMPT were confirmed *in vivo* (214, 220, 223).

Elevated eNAMPT levels may play an important role in promoting tumor cell migration and invasion. For example eNAMPT was shown to stimulate the migration of breast cancer, osteosarcoma, ovarian cancer, chondrosarcoma, and prostate cancer cells *in vitro* (214, 224–227). In colorectal cancer (CRC) cells, eNAMPT upregulated the expression of stromal-derived factor 1, a chemokine known to stimulate CRC cell migration (228). Importantly, a neutralizing anti-NAMPT antibody was able to successfully inhibit prostate cancer cell invasiveness in a mouse orthotopic xenograft model (229). As demonstrated by Soncini et al. (75), one of the mechanisms by which eNAMPT could promote cancer cell migration and metastasis is by inducing epithelial-to-mesenchymal transition (EMT) via transforming growth factor β signaling. eNAMPT could also trigger EMT via NF-κB/Snail signaling, as shown by others (230, 231). Additional mechanisms proposed to explain the pro-migratory and pro-invasive effects of eNAMPT are 1) the induction of matrix-degrading enzymes in cancer cells, such as MMP-2 and -9 gelatinases (214, 218, 221, 222, 226, 227, 232) and 2) the stimulation of angiogenesis by inducing pro-angiogenic factors in endothelial cells (81) and cancer cells (222).

Importantly, eNAMPT may contribute to cancer progression by fostering immunosuppression in the tumor micro-environment. For instance, studies reported that eNAMPT induced the polarization of macrophages towards the pro-tumorigenic M2 phenotype (63, 71). eNAMPT was also shown to boost the secretion of immunosuppressive, tumor-promoting factors such as indoleamine 2,3-dioxygenase, CCL18, IL-10, IL-1β, IL-6, and CXCL8 (63, 66). Audrito et al. reported that eNAMPT may contribute to immunosuppression in chronic lymphocytic leukemia by inducing the differentiation of monocytes into a specialized class of leukemia-associated macrophages called nurse-like cells that create an immunosuppressive niche to promote cancer cell survival and inhibit T cell proliferation (63).

Last but not least, *in vitro* studies suggest that eNAMPT could mediate resistance to cancer therapies. A recent study revealed that eNAMPT, by boosting thymidylate synthase expression in cancer cells, reduced the sensitivity of CRC to capecitabine (233). Likewise, eNAMPT was shown to induce the phosphorylation of estrogen receptor α, contributing to breast cancer resistance to tamoxifen (234, 235). Finally, the fact that eNAMPT enhances the antioxidant capacity of tumor cells may also contribute to their resilience and drug resistance (217, 236).

#### 5.6.2 Clinical studies

Numerous studies of samples obtained from cancer patients assessed the association between circulating eNAMPT levels on the one hand, and cancer size/stage, patient survival or specific biomarkers, on the other. As summarized in **Table 3** and as reviewed by Dalamaga et al. (213), elevated levels of eNAMPT are present in cancer patients, positively correlating with tumor size and cancer stage (23, 214, 219, 227, 232, 239, 241–246). eNAMPT levels in cancer patients could also be (negatively) correlated with disease-free and overall survival and (positively) with lymph node invasion and/or metastasis (214, 232, 245, 247). As an example, elevated eNAMPT levels were established as an independent risk factor for myometrial invasion in uterine cancer (248). Circulating eNAMPT levels also correlated with cancer biomarkers such as CA 15-3 in breast cancer (247), alpha-fetoprotein in hepatocellular carcinoma (243), and lactate dehydrogenase in metastatic melanoma (23). On a separate note, melanoma patients with Programmed death-ligand 1-positive (PD-L1^+^) lesions had significantly higher plasma eNAMPT concentrations than patients with PD-L1^−^ lesions, hinting at a link between eNAMPT and the tumor-associated inflammatory response (23).

**Table 3:** eNAMPT as a potential biomarker in cancer.

Author	Year	Sample size	Results
**Sawicka-Gutaj et al. (** 237)	2022	22 patients with adrenocortical carcinomas 26 patients with benign adrenocortical tumors	Serum eNAMPT levels were significantly higher in patients with adrenocortical carcinomas than in patients with benign adrenocortical tumors (P = 0.003). Furthermore, eNAMPT concentrations were higher in clinically advanced patients with metastases than in other patients (P = 0.022). Finally, a ROC analysis showed that serum eNAMPT levels could be used to distinguish between patients with adrenocortical carcinomas and those with benign tumors (sensitivity = 50%, specificity = 92.3%, AUC = 0.739, P = 0.001).
**Pazgan-Simon et al. (** 238)	2020	69 HCC patients 20 controls	Serum eNAMPT concentrations were significantly elevated in HCC patients compared with healthy controls (P = 0.04).
**Sun et al. (** 227)	2020	34 PCa patients 130 high-risk subjects without PCa 105 non-age-matched controls 27 age-matched controls	Plasma eNAMPT levels of PCa patients were significantly higher than those of high-risk subjects without PCa (P < 0.05), non-age-matched controls (P < 0.01), or age-matched controls (P < 0.001). eNAMPT concentrations were significantly elevated in extraprostatic, invasive PCa than in organ-confined PCa (P = 0.028). In line with this, plasma eNAMPT levels significantly correlated with PCa tumor stage (P = 0.041). A ROC analysis revealed that plasma eNAMPT levels could be used to diagnose PCa (sensitivity = 79%, specificity = 82%, AUC = 0.79, P < 0.05).
**Audrito et al. (** 23)	2018	163 BRAF-mutated melanoma patients (113 metastatic melanoma, 50 localized melanoma) 38 controls	Higher plasma eNAMPT levels were found in patients with metastatic melanoma than in those with localized stage I-II melanoma or controls (P ≤ 0.0001). In addition, eNAMPT levels correlated positively with LDH levels, a marker of tumor burden, in 39 patients (r = 0.41, P = 0.008). Melanoma patients exhibiting PD-L1^+^ lesions had significantly increased plasma levels of eNAMPT compared with patients with PD-L1^−^ lesions (P ≤ 0.01).
**Cymbaluk-Ploska et al. (** 239)	2018	78 patients with endometrial cancer 63 controls	In patients with endometrial cancer, serum eNAMPT concentrations were significantly higher than in controls with a normal endometrium (P = 0.002). Furthermore, eNAMPT levels were higher in patients with highly advanced cancer compared with those with less advanced cancers (P = 0.0002), as well as in G3 versus G2 grading cancers (P = 0.002), in patients with > 2-cm melanoma versus those with < 2-cm melanoma (P = 0.003), and in patients with deep myometrium infiltration versus those with superficial infiltration (P = 0.004). In line with this, in patients with endometrial cancer, high levels of eNAMPT correlated with shorter overall survival (P = 0.0001). Finally, a ROC analysis showed that eNAMPT levels could be used to distinguish between patients with endometrial cancer and individuals with benign endometrial lesions (AUC = 0.89, 95% CI = 0.74–0.9, P < 0.002).
**Sowa et al. (** 240)	2018	59 patients with parotid gland tumor (30 PA, 21 WT, 8 ACC) 30 controls	Significantly higher serum eNAMPT levels were found in patients with parotid gland tumors (PA, WT, and ACC) than in healthy controls (P < 0.05).

Previously reviewed by Dalamaga et al. (213). ACC, acinic cell carcinoma; AUC, area under the curve; CI, confidence interval; eNAMPT, extracellular nicotinamide phosphoribosyltransferase; HCC, hepatocellular carcinoma; LDH, lactate dehydrogenase; PA, pleiomorphic adenoma; PCa, prostate cancer; PD-L1, programmed cell death-ligand 1; ROC, receiver operating characteristic; WT, Warthin’s tumor.

As a corollary, eNAMPT may represent an appealing novel therapeutic target in oncology, given its proposed implication in cancer pathogenesis and progression. In any case, eNAMPT prospects as a prognostic/predictive biomarker look particularly encouraging. Several studies demonstrated that eNAMPT could rather accurately predict cancer progression (227, 237, 239, 247) and a recent meta-analysis revealed a correlation between eNAMPT levels and cancer risk (21).

## 6 eNAMPT as a potential drug target

Historically, therapeutic treatment approaches have focused on NAMPT enzymatic activity and several low molecular weight NAMPT enzyme inhibitors (NAMPTi) are available (5, 249, 250). Among these, FK866 (also named APO866, (E)-Daporinad, and WK1) and GMX1778 (also named CHS-828) were among the first to be synthetized and tested. These “early” NAMPTi usually show potent anti-cancer activity in preclinical models, but could not progress beyond early clinical trials due to poor efficacy associated with dose-limiting toxicities such as thrombocytopenia and gastrointestinal toxicity (5, 249). In an attempt to broaden the therapeutic index, a second generation of NAMPTi (such as OT-82), as well as dual-function enzymatic inhibitors (i.e., inhibiting NAMPT enzymatic activity plus another target overexpressed in tumors, e.g., KPT-9274, which also blocks P21-activated kinase 4), were generated and are currently being evaluated in phase I clinical trials in cancer (250).

Concerning eNAMPT, its proposed pathogenic functions are typically not contingent on enzymatic activity, and thus cannot be inhibited by NAMPTi. Antibodies, in contrast, are well suited for the neutralization of extracellular factors like eNAMPT, and several *in vitro* and *in vivo* studies already explored this therapeutic strategy (summarized in **Tables 4**, **5**). For example, Kieswich et al. successfully used a commercial anti-NAMPT polyclonal antibody *in vivo* to improve the diabetic phenotype of HFD-fed mice, pancreatic islet function, glycemic control, and insulin resistance (130). Another example is provided by Colombo et al., who developed a NAMPT-neutralizing mAb capable of limiting acute and chronic colitis in experimental mouse models (188). Unquestionably, the most advanced eNAMPT inhibitor is currently ALT-100, a humanized mAb developed by Joe G.N Garcia and coworkers. Since ALT-100 is cross-reactive with NAMPT from multiple mammalian species, it could be tested in mice but also in several non-murine *in vivo* models. Hence, ALT-100 demonstrated to reduce the severity of murine and porcine inflammatory lung injury (205–207), pulmonary hypertension in rats (255), radiation-induced lung fibrosis in mice (257), and prostate cancer cell proliferation, invasion, and metastasis (229). More recently, ALT-100 has also shown therapeutic efficacy in mouse models of lung vasculitis/hemorrhage, nonalcoholic fatty liver disease and intra-amniotic inflammation (190, 258, 259). ALT-100 is currently completing a first-in-human study in healthy volunteers (NCT05426746).

**Table 4:** Preclinical *in vitro* studies assessing the neutralizing effect of anti-NAMPT antibodies.

Author	Year	Cells	Treatment	Results
**Pillai et al.** (251 **).**	2013	Neonatal rat cardiomyocytes	Anti-NAMPT pAb (Lampire Biological Laboratories (178))	Anti-NAMPT pAb blocked the eNAMPT-induced increase in leucine incorporation into cardiomyocyte proteins (P < 0.001) and the increase in cardiomyocyte size (P < 0.001), indicating that the antibody prevented the prohypertrophic effect of eNAMPT.
**Audrito et al. (** 63)	2015	Nurse-like cells	Anti-NAMPT pAb (Lampire Biological Laboratories (178))	eNAMPT-induced STAT3 signaling activation was reduced by the anti-NAMPT pAb treatment of NLCs (P = 0.02).
**Liu et al. (** 252)	2015	NCI-H446 cells	Anti-NAMPT antibody (Santa Cruz Biotechnology)	Neutralization of eNAMPT inhibited the transendothelial migration of NCI-H446 cells (P < 0.01).
**Zhang et al. (** 253)	2018	Macrophages isolated from human PBMC, and polarized into M2 phenotype	Anti-NAMPT antibody (LifeSpan Biosciences)	Treatment of M2 macrophages treatment with an anti-NAMPT antibody reduced their CD206 expression levels as well as IL-1Ra and IL-10 release (P < 0.05).
**Colombo et al. (** 188)	2020	4T1 cells	Anti-NAMPT mAb C269	The C269 antibody reduced eNAMPT-induced STAT3 phosphorylation in 4T1 cells in a dose-dependent manner.
**Quijada et al. (** 206)	2020	Human lung ECs	Anti-NAMPT pAb or ALT-100 anti-NAMPT mAb	An anti-NAMPT pAb reduced the activation of the eNAMPT-induced NF-κB and MAPK signaling pathways in human lung ECs (P < 0.05). Both the anti-NAMPT pAb and mAb ALT-100 reduced the eNAMPT-induced breakdown in EC barrier integrity (determined by measuring the transendothelial electrical resistance of ECs) (P < 0.05).
**Sun et al. (** 229)	2021	DU145 and PC3 cells	ALT-100 anti-NAMPT mAb	Treatment of PCa cells with ALT-100 significantly reduced eNAMPT-induced NF-κB phosphorylation (P < 0.05).
**Xiao et al. (** 254)	2021	A549 and H1299 cells	Anti-NAMPT pAb A300-778, (Invitrogen)	The anti-NAMPT pAb inhibited the radiation-induced migration and invasion of A549 cells (P < 0.01). In addition, the antibody reversed the radiation-mediated upregulation of Snail in A549 and H1299 cells (P < 0.01).
**Colombo et al. (** 32)	2022	Murine PECs	Anti-NAMPT mAb C269	Anti-NAMPT C269 reduced the eNAMPT-mediated upregulation of inflammatory M1-related gene expression (*Cox2, Nos2, IL-1β, Cxcl9, Cxcl10*, and *IL-6*) in PECs (P < 0.05).

CD206, cluster of differentiation 206; Cox2, cyclooxygenase-2; Cxcl, chemokine C-X-C motif ligand; EC, endothelial cell; eNAMPT, extracellular nicotinamide phosphoribosyltransferase; IL, interleukin; IL-1Ra; interleukin-1 receptor antagonist; mAb, monoclonal antibody; MAPK, mitogen-activated protein kinase; NF-κB, nuclear factor kappa-light-chain-enhancer of activated B cells; NLC, nurse-like cells; NOS2, nitric oxide synthase 2; pAb, polyclonal antibody; PBMC, peripheral blood mononuclear cell; PCa, prostate cancer; PEC, peritoneal macrophages; STAT3, signal transducer and activator of transcription 3.

**Table 5:** Preclinical *in vivo* studies evaluating the therapeutic potential of anti-NAMPT antibodies.

Author	Year	Disease model	Treatment (dose/administration)	Results
**Hong et al. (** 208)	2008	Murine model of VILI	Anti-NAMPT pAb from Lampire Biological Laboratories	Anti-NAMPT pAb reduced the VILI-mediated BAL PMN accumulation and tissue albumin leakage in mice (P < 0.05). The eNAMPT-induced accumulation of BAL PMNs was also diminished in the presence of this antibody (P < 0.05).
**Kieswich et al. (** 130)	2016	Mouse model of diabetes (HFD-fed mice)	Anti-NAMPT pAb from LifeSpan BioSciences	Administration of the anti-NAMPT antibody reversed the diabetic phenotype of HFD-fed mice, reduced β-cell dysfunction (increased insulinemia and islet size; P < 0.05) and improved tissue and systemic inflammation (reduced expression of IL-1β, TNF-α, CCL2 in isolated islets; P < 0.01).
**Colombo et al. (** 188)	2020	DNBS- and DSS-induced colitis mouse models	C269 anti-NAMPT mAb	C269 antibody reduced body weight loss (DNBS-treated mice, P < 0.001), colon shortening (DNBS and DSS, P < 0.001), transmural necrosis, and oedema (assessed by histological analysis, DNBS and DSS, P < 0.05).
**Quijada et al. (** 206)	2021	“One-hit” (LPS) and “two-hit” (LPS/VILI) ARDS mouse models	Anti-NAMPT pAb and ALT-100 anti-NAMPT mAb	Both the anti-NAMPT pAb and ALT-100 reduced LPS-induced histological evidence of inflammation and injury, as well as BAL protein content and BAL PMN counts, in preclinical “one-hit” and “two-hit” ARDS mouse models (P < 0.05). In addition, ALT-100 decreased plasma IL-6 levels in mice exposed to “one-hit” or “two-hit” challenge (P < 0.05).
**Sun et al. (** 229)	2021	PCa orthotopic xenograft mouse models	ALT-100 anti-NAMPT	The ALT-100 antibody elevated the probability of survival of SCID mice bearing orthotopic DU145 or PC3 xenografts (P < 0.05). In addition, ALT-100 induced a reduction in DU145 and PC3 tumor size (P < 0.05).
**Ahmed et al. (** 255)	2021	PAH hypoxia/Sugen rat model	ALT-100 anti-NAMPT mAb	ALT-100 reduced plasma eNAMPT levels, plasma IL-6 levels, plasma TNF-α levels, vessel wall thickness, RVSP, and RV/(S+LV) ratio, in rats exposed to hypoxia/Sugen (P < 0.05).
**Garcia et al. (** 256)	2021	WTLI murine model of radiation pneumonitis	Anti-NAMPT pAb or ALT-100 anti-NAMPT.	Anti-NAMPT pAb and ALT-100 reduced WTLI-induced histologic inflammatory injury, BAL protein levels, BAL cell counts, and the RILI severity score in WTLI 20-Gy-exposed mice (P < 0.05). In addition, ALT-100 reduced the plasma levels of eNAMPT, IL-6, and IL-1β in these animals (P < 0.05).
**Bermudez et al. (** 205)	2022	“One-hit” (LPS) and “two-hit” (LPS/VILI or blast trauma/VILI) ARDS rat models	Anti-NAMPT pAb or ALT-100 anti-NAMPT mAb	Both anti-NAMPT pAb and ALT-100 reduced LPS-induced histological evidence of inflammation and injury, BAL protein content, and BAL PMN counts in preclinical “one-hit” (LPS) and “two-hit” (LPS/VILI) ARDS rat models (P < 0.05). In the “two-hit” (blast-trauma/VILI) rats, anti-NAMPT pAb diminished histological evidence of inflammation and injury compared to PBS (P < 0.05).
**Sammani et al. (** 207)	2022	ARDS/VILI porcine model	ALT-100 anti-NAMPT mAb	The ALT-100 antibody reduced total BAL cell and BAL PMN counts, the lung tissue wet/dry weight ratio, the plasma levels of IL-6, IL-1Ra, and Ang-2, and BAL protein and lung tissue albumin levels in LPS/VILI-challenged pigs (P < 0.05). In addition, ALT-100 attenuated acute kidney injury in LPS/VILI-challenged pigs, as evidenced by reductions in histopathologic score, caspase-3 cleavage staining levels, and plasma lipocalin levels (P < 0.05).
**Garcia et al. (** 257)	2022	WTLI murine model of radiation fibrosis	Anti-NAMPT pAb or ALT-100 anti-NAMPT mAb	Both the anti-NAMPT pAb and ALT-100 mAb significantly reduced WTLI-mediated histologic features of lung injury, BAL protein levels, and BAL cell counts, compared with the PBS/IgG1 control (P < 0.05). In addition, the ALT-100 mAb significantly decreased trichrome blue staining (an indicator of lung fibrosis) and plasma IL-1β levels (P < 0.05).
**Tumurkhuu et al. (** 190)	2022	Murine model of pristane-induced lung vasculitis and diffuse alveolar hemorrhage	ALT-100 anti-NAMPT mAb	The ALT-100 antibody significantly reduced immune cell infiltration into the lung perivascular area of pristane-challenged mice (P < 0.05). In the same mice, ALT-100 significantly attenuated the pristane-induced increase in BAL cell counts and protein levels, as well as pristane-induced NF-κB inflammatory signaling activation (P < 0.05).
**Sun et al. (** 258)	2023	Streptozotocin (STZ)/HFD murine NAFLD model	ALT-100 anti-NAMPT mAb	The treatment of STZ/HFD mice with the ALT-100 antibody reduced NASH severity, as evidenced by the reduction in steatosis score, hepatic triglyceride levels, and NAFLD activity score in mice (P < 0.05). In addition, eNAMPT neutralization by ALT-100 also decreased plasma TNF-α levels and hepatic Snail expression in the same animals (P < 0.05).
**Ahmed et al. (** 259)	2023	Murine model of intrauterine inflammation	ALT-100 anti-NAMPT mAb	eNAMPT neutralization with ALT-100 allowed to significantly reduce preterm birth from LPS-exposed dams and to improve pup survival (P < 0.05). Furthermore, ALT-100 significantly decreased plasma IL-6, KC and MCP-1 levels in LPS-challenged dams (P < 0.05).

Ang-2, angiopoietin-2; ARDS, acute respiratory distress syndrome; BAL, bronchoalveolar lavage; CCL-2, chemokine (C-C motif) ligand 2; DNBS, dinitrobenzene sulfonic acid; DSS, dextran sulfate sodium; eNAMPT, extracellular nicotinamide phosphoribosyltransferase; HFD, high fat diet; HOMA-IR, homeostatic model assessment of insulin resistance; IL, interleukin; IP, intraperitoneally; IT, intratracheally; IV, intravenously; KC, keratinocyte-derived cytokine; LPS, lipopolysaccharide; mAb, monoclonal antibody; MCP-1, monocyte chemoattractant protein-1; NAFLD, non-alcoholic fatty liver disease; NASH, non-alcoholic steatohepatitis; pAb, polyclonal antibody; PBS, phosphate-buffered saline; PCa, prostate cancer; PAH, pulmonary arterial hypertension; PMNs, polymorphonuclear neutrophils; QUICKI, Quantitative Insulin Sensitivity Check Index; RILI, radiation-induced lung injury; RVSP, right ventricular systolic pressure, RV/(S + LV), ratio of the weight of the right ventricle to that of the septum plus left ventricle; SCID, severe combined immunodeficiency; STZ, streptozotocin; TNF-α, tumor necrosis factor α; VILI, ventilator-induced lung injury; WTLI, whole thoracic lung irradiation.

Recently, a novel therapeutic approach for targeted degradation of intracellular NAMPT was made possible thanks to the development of the “molecular glue” technology (PROTAC) (260). A mechanistic consequence of lowering intracellular NAMPT protein levels is the concomitant reduction of eNAMPT in the extracellular space. Thus, PROTAC is expected to limit both the enzymatic (principally intracellular) and non-enzymatic (extracellular) activities of NAMPT. Indeed, some promising preliminary results have been obtained with this class of molecules *in vitro* and *in vivo* (261, 262). One study reported that NAMPT-specific PROTACs outperformed FK866 in terms of tumor-killing activity (261). Strikingly, the authors showed that, unlike FK866, PROTACs were able to inhibit the NF-κB and MAPK/ERK1/2 pathways, implying that they also prevent eNAMPT pro-inflammatory DAMP activities (261). Notwithstanding these encouraging results, further studies are needed to determine whether NAMPT-targeting PROTACs could be developed into safe and efficacious drugs for patients.

## 7 Conclusions and perspectives

Numerous clinical studies, mostly published in the past decade, have reported a correlation between elevated levels of circulating eNAMPT and diverse metabolic and inflammatory disorders, presenting physicians with an opportunity of using eNAMPT levels as a disease activity indicator, prognostic biomarker, or even as a patient stratification tool. There is also mounting, well-documented, preclinical evidence suggesting that elevated eNAMPT levels may contribute to disease pathophysiology by exerting pleiotropic and systemic effects on metabolism and immunity via DAMP/adipocytokine/pro-angiogenic factor activities. Consequently, therapeutic approaches for eNAMPT inhibition, principally with mAbs, are now being developed and tested in various disease models. The leading experimental drug, mAb ALT-100, is in early clinical development, so we may soon learn more about the potential of eNAMPT as therapeutic target.

All that said, while eNAMPT may be a pleiotropic factor implicated in disease pathophysiology, it is also ubiquitously secreted and present at low physiological levels in healthy individuals, and the benefits of physiological eNAMPT release are not fully appreciated, aside from specific examples, such as maintaining pancreatic beta cell function. Likewise, little is known about how eNAMPT physiological levels in tissues and in the circulation are affected by diverse environmental and physiological cues, by age, activity, etc. Other aspects of eNAMPT biology remain unclear as well, in particular the exact modalities of eNAMPT interaction with target cells and its purported cell surface receptors. We hope that future research will help shed light on the currently unknown aspects of this fascinating protein.

## Author contributions

ES: Conceptualization, Investigation, Writing – original draft, Writing – review & editing, Visualization. AN: Writing – review & editing, Visualization. KM: Conceptualization, Project administration, Supervision, Visualization, Writing - review & editing.
